# Freeway ramp metering based on PSO-PID control

**DOI:** 10.1371/journal.pone.0260977

**Published:** 2021-12-09

**Authors:** Junjun Wei, Kejun Long, Jian Gu, Zhengchuan Zhou, Shun Li

**Affiliations:** 1 School of Traffic & Transportation Engineering, Changsha University of Science and Technology, Changsha, China; 2 Hunan Key Laboratory of Smart Roadway and Cooperative Vehicle-Infrastructure Systems, Changsha University of Science and Technology, Changsha, China; University of Shanghai for Science and Technology, CHINA

## Abstract

Ramp metering on freeway is one of the effective methods to alleviate traffic congestion. This paper advances the field of freeway ramp metering by introducing an application to the on-ramp, capitalizing on the macro traffic follow theory and improved the freeway traffic flow. The Particle Swarm Optimization (PSO) based on Proportional Integral Derivative (PID) controller is further developed to single ramp metering as well as to optimize the PID parameters. The approach is applied to a case study of the Changyi Freeway(G5513) in Hunan, China. The simulation is conducted by applying the actual profile traffic data to PID controller to adjust the entering traffic flow on the freeway on-ramp. The results show that the PSO-PID controller tends to converge in about 80 minutes, and the density tends to be stable after 240 iterations. The system has smaller oscillation, more accurate adjustment of ramp regulation rate, and more ideal expected traffic flow density. The traffic congestion on mainline is effectively slowed down, traffic efficiency is improved, and travel time and cost are reduced. The nonlinear processing ability of PSO-PID controller overcomes the defects of the traditional manual closing ramp, and can be successfully applied in the field of intelligent ramp metering.

## 1. Introduction

The aim of freeway on-ramp metering is to control the traffic demand. According to the traffic state of the main line, the ramp entering rate can be adjusted to support the traffic flow distribution of main line more regular, and the traffic capacity can be maximized. On-ramp metering includes ramp metering and ramp closure. According to the traffic information report of Changyi Freeway in March and April of 2018, it can be seen that the days of traffic congestion in March was 19 days, and the times of traffic control was 16. Most of the time in China, the treatment of freeway congestion is to close the ramp through traffic control, and the closing time is usually very long. Ramp closure will not only affect journeys for travelers, but also may trigger accidents. Only relying on the observation and feedback of the road operation state and closing the ramp cannot completely solve the problem of freeway congestion. Therefore, it is necessary to develop the intelligent control of freeway ramp metering.

In this paper, according to the nonlinear and time-varying characteristics of freeway traffic system, we take the measure of leveraging on a fuzzy automatic particle swarm optimization (PSO) based PID controller to improve the on-ramp entering flow rate to main line. The main contribution of this study lies in proposing the traffic flow state model according to the macro traffic flow theory, and developing the fuzzy automatic PID controller to optimize the traffic flow for ramp metering. The ramp regulation rate is determined by the controller, and the parameters are adjusted by fuzzy logic according to the density tracking error and the change of error. Combined with the nonlinear feedback theory, a fuzzy PSO-PID ramp controller is designed to deal with the expressway traffic flow density problem of the macro traffic flow model. According to the real-time traffic state, the parameters are dynamically adjusted by PSO method to obtain the minimum density tracking error. The principle of PID control system is simple, and has high robustness and good adaptability in nonlinear control. This method has the functions of self-organization, evolution, and memory. The control quality is obviously better than the traditional PID controller, and the response speed is obviously faster than the BP neural network based PID controller. The PSO-PID control provides a scientific strategy for the freeway on-ramp metering.

This remainder of the paper is organized as follows. Section 2 reviews relevant papers in the literature and highlights the research gap. Section 3 provides a quantitative way to estimate the traffic state by using profile data, and develops the optimized PID framework based on PSO algorithm. A case study simulation based on Changyi Freeway (G5513) is conducted in Section 4. Finally, Section 5 draws discussion and conclusion, as well as suggests future research directions.

## 2. Literature review

The basic principle of ramp metering is to effectively regulate the upstream and downstream traffic volume of the main line at the on-ramp by limiting the traffic flow into the freeway, so as to ensure that the traffic demand on the main line does not exceed its traffic capacity. According to the control range, ramp control can be divided into local / isolated metering and coordinated metering; according to the real-time response, ramp control can be divided into fixed-time strategies and responsive control [1]. The basic idea of the on-ramp timing control method proposed by Watleworth in 1965 is to estimate the fixed mediation rate of different time periods based on historical traffic data [2]. Its disadvantage is that it does not consider the time-varying characteristics of traffic conditions and cannot adjust the ramp inflow rate according to the real-time traffic. Inductive ramp control is based on the real-time parameters, with a certain control parameter kept near the optimal value as the goal, to calculate the on-ramp mediation rate. Early control algorithms include demand-capacity control and occupancy control. These methods belong to open-loop control, which is sensitive to external disturbance and has poor robustness [3]. In 1990s, Papageorgiou proposed ALINEA method, which is a feedback control method based on classical automatic control theory. The ramp metering is regarded as a state regulator by comparing the difference between the detection state and the target state, the ramp inflow rate is adjusted to maintain the density / occupancy rate of the downstream mainline at an ideal state. This is a typical single local ramp metering strategy, which is widely used in engineering because of its simple, flexible and effective characteristics. Papageorgiou proposed the METALINE method later for the overall control of freeway on-ramp, which is the extension of ALINEA method. These algorithms can make timely response to traffic conditions, and the control effect is improved compared with the timing algorithm, but the learning robustness is still insufficient [4–7]. Meanwhile, the traditional ramp metering method requires that the nonlinear partial differential equation model be simplified into a linear difference equation model, which will affect the control performance. Therefore, some researchers turn to use fuzzy control, artificial neural network and expert system to design intelligent controller. Taylor C (1997) and Zhang (1998) applied heuristic algorithms such as fuzzy control and artificial neural network to single dynamic ramp metering, and achieved certain results [8,9]. Liang (2005) et al. presented a nonlinear method to design the feedback controller of freeway on-ramp. The nonlinear feedback controller consists of freeway traffic flow model and proportional-integral regulator. However, the parameters cannot be adjusted online [10–15]. Bellemansa (2006) et al. proposed a ramp metering predictive method based on real traffic data [16]. Compared with the classical ALINEA, the results show that the model performs better than the ALINEA during the early peak period. Zeng (2007) studied the RBF neural network tuning PID controller parameters, and applied to the freeway on-ramp control. The simulation results showed that the controller has excellent dynamic and steady performance [17]. Loannis (2010) et al. proposed a feedback control strategy HERO (Heuristic Ramp-metering Coordination) [18]. The control strategy of single ramp uses ALINEA, while the coordinated control of multi-ramp is coordinated by the control center. Fares A. (2014) et al. proposed an intelligent Q-learning algorithm based on Markov model for freeway congestion control [19–22]. This algorithm has reward and evaluate function by collecting and analyzing past data. In the view of certain limitations to ramp metering, Meshkat (2015) et al. applied a prioritized control strategy to distribute traffic flow more rationally across the entire road network [23]. For the current difficulties in controlling discrete partial differential equation systems, Belletti (2017) et al. designed a new multi-intelligent parametric-free control algorithm, and showed how to realize parametric-free control based on BP neural network [24–27]. In addition, a more accurate BeATS simulator is adopted by researchers, which is the most advanced parameter control system compared with ALINEA, and achieved comparable control effect. Traffic estimation and simulation are the basis of freeway ramp metering, on this issue, Long (2018, 2019) and Gao (2019) et al. conducted traffic estimation and prediction of traffic operating efficiency using the multiple source data [28–38].

Basically, for a single ramp, on-ramp control can be divided into two categories: timing control and traffic sensing control. The timing control is based on the historical traffic volume data according to the preset regulation rate, so it cannot respond to the random changes of traffic volume, and it is difficult to eliminate traffic congestion. Traffic sensing control is based on the real-time traffic volume detection data to determine the ramp adjustment rate. It is superior to timing control in preventing and reducing traffic jams. The existing control strategies and methods seem to be very systematic and comprehensive, which can solve the problems of the whole system, but in fact they are very idealized. When the acquisition data is incomplete, part of the detector does not work, or the detection data is missing, those models that need to rely on complete data acquisition have limited control effect on the system. Furthermore, freeway traffic flow is a complex system, due to the traffic flow detector is limited and sparse, it is unable to obtain the parameters of traffic and state, not only the complete state in space, but also the complete information in parameters. The changeable traffic state leads to the traffic flow fluctuation and emergency events, which makes it very difficult to predict.

In this paper, a nonlinear feedback method is proposed to design an on-ramp traffic sensing controller, and the controller is simulated. To this end, this research will analyze the real profile traffic data, and provide the proper ramp metering method to Chinese freeway.

## 3. Methodology

### 3.1 Introduction of profile traffic data

The detection of profile traffic flow is the main method currently used in China’s freeways. The main technical approach of collecting traffic data is usually through various detection devices, such as loop detector, microwave detector, video detector, etc. The detection frequency can be 30 seconds, 1 minute, 5 minutes and 15 minutes. Through the collection of real-time data, such as density, speed, traffic volume, and travel time, these data are sent to the background system for storage and analysis. Thus, the road traffic status can be judged, predicted and timely processed. The data collected in this paper comes from the Changyi Freeway(G5513), mainly includes the data of main line and toll station. The main line contains the traffic flow data on different lanes, vehicle type, and location of detection points in every 5 minutes. These data can be used to estimate the traffic flow state of the main line. The toll station data covers the detailed running data of each ramp toll station, including date, vehicle license number, ramp number of entry and exit, time of entry and exit, etc. The number of vehicles entering and leaving the main line through the ramp can be counted through such information. Therefore, the travel time and travel route can be estimated according to the vehicle license and the access time.

Most of the traffic flow data are collected from the ramp of Changyi Freeway. The closed ramp flow data between different interchanges can be counted by classifying the data in March according to the number of toll station. It can be seen that the Ningxiang toll station near the city end is indeed a key section of the expressway flow based on the data analysis. This is consistent with the freeway blocking information from traffic police for closing ramp with high frequency on holidays and daily peak hours.

### 3.2 Macro traffic flow model at freeway on-ramp

**Fig 1:**
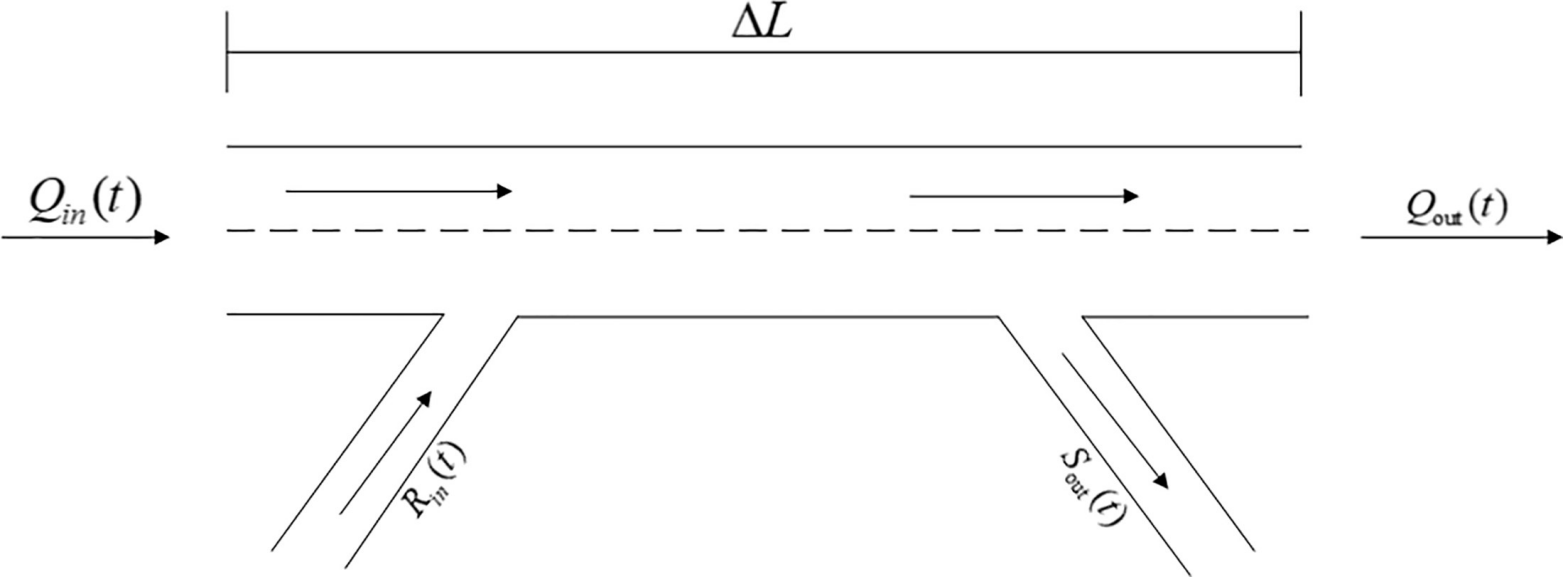
Freeway weave section with on/off ramp schematic diagram.

The macro traffic flow model regards the traffic flow as a compressible continuous fluid medium composed of massive vehicles, and studies the average behavior of vehicle groups from the perspective of Hydrodynamics. It is usually focus on the overall state of traffic flow, density, and speed. The data collected by Changyi Freeway conform to the macro traffic flow model indexes. Therefore, this paper applies the macro traffic flow model to analyze the traffic state of the freeway. Fig 1 is a freeway weave section with on/off ramp schematic diagram.

According to the flow conservation principle, it is known that:

$$N(t+1)=N(t)+\theta \Delta t[Q_{in}(t)-Q_{out}(t)]+\Delta t[R_{in}(t)-S_{out}(t)]$$ (1)

Here, *Q*_*in*_*(t)* is entering traffic flow on road section at time *t*; *Q*_*out*_*(t)* is outgoing traffic flow; *R*_*in*_*(t)* is entering traffic flow on-ramp; *S*_*out*_*(t)* is outgoing traffic flow on-ramp; *θ* is number of lanes; and *ΔL* is section length. *N(t+1)* is traffic flow in the *ΔL* section at time *(t+1)*; *N(t)* is traffic flow in the *ΔL* section at the previous time. According to the definition of density:

$$\rho (t)=\frac{N(t)}{\theta \Delta L}$$ (2)

Bring Eq 2 into Eq 1:

$$\rho (t+1)=\rho (t)+\frac{\Delta t}{\Delta L}[Q_{\mathrm{in}}(t)-Q_{out}(t)]+\frac{[R_{\mathrm{in}}(t)-S_{out}(t)]\Delta t}{\theta \Delta L}$$ (3)

From the flow-density relation, it can be seen that:

$$q(t)=V_{f}[\rho (t)-\frac{\rho^{2}(t)}{\rho_{jam}}]$$ (4)

Bring Eq 4 into Eq 5:

$$\rho (t+1)=\rho (t)+\frac{\Delta t}{\Delta L}\{Q_{in}(t)-V_{f}[\rho (t)-\frac{\rho^{2}(t)}{\rho_{jam}}]\}+\frac{[R_{in}(t)-S_{out}(t)]\Delta t}{\theta \Delta L}$$ (5)

Here, $\frac{\Delta t}{\Delta L}$ is the constant; *Q*_*in*_*(t)* is obtained by road detector; *Δt* is monitoring duration; *V*_*f*_ is free-flow speed; *ρ(t)* is the state variable; *ρ*_*jam*_ is jamming density. Eq 5 describes the traffic flow process of a freeway section, the initial state density of the system can be set and iteratively calculated in the control system. Therefore, Eq 5 can only be considered as a function of density and ramp entrance adjustment rate. In the following section 3.3, this model will be applied into the PID controller.

### 3.3 Single Ramp metering based on PSO-PID control

- **Objective of ramp control**. According to the traffic flow theory, traffic density is the key parameter to reflect the traffic congestion level. If the traffic density exceeds the critical density, the traffic will become very crowded. Although the on-ramp flow is reduced, the congestion still needs a long time to return to normal. Therefore, the goal of ramp metering is to guarantee the traffic density not exceed the critical density, and generally maintain the density in the negative neighborhood of the critical density, that is, *ρ*_*d*_ *= ρ*_*c*_*-ɛ*, where *ρ*_*d*_ is the expected density, *ρ*_*c*_ is the critical density, and *ɛ* is an appropriate small positive number.
- **Design of PSO-PID controller**. Nowadays, with the rapid development of computer technology, many new control methods have emerged, but the classical closed-loop control theory of PID is still widely used in engineering field at present because of its simple principle and good adaptability. The PID control is a kind of feedback control, which is based on the input and output control of traffic flow. Although it cannot have a very clear grasp of all sections and spaces of the whole expressway, it can control the relationship between their input and output according to the state of some important sections, such as the merging point of on-ramp, the diverging point of off ramp and some important sections. The PID control is concerned with the variables and compared to the expected values to correct and control the system response. It has a complete set of parameters setting and design rules, which can be easily mastered by freeway policemen.

However, the parameters of PID cannot be adjusted in real time. Particle swarm optimization (PSO) is a method to achieve the optimal solution by interacting information within the population, and it provides a new idea to control the traffic demand. According the macro traffic flow theory, the freeway traffic flow estimation method based on profile data is proposed. The intelligent adaptive PSO-PID controller for freeway on-ramp is introduced into the ramp metering.

The traditional PID control method needs the accumulation of all the errors in the calculation, and the calculation is massive and complicated. Therefore, the PID control method applied in this paper is the incremental PID control based on PSO algorithm. It is a kind of on-line adaptive PID controller with good adaptability and robustness. The output of incremental PID control is *Δu(k)*. With regard to the above formula subtraction, the incremental expression is obtained:

Δu(k)=u(k)−u(k−1) (6)

$$\Delta u(k)=K_{P}(error(k)-error(k-1))+K_{I}error(k)+K_{d}(error(k)-2error(k-1)+error(k-2))$$ (7)

**Fig 2:**
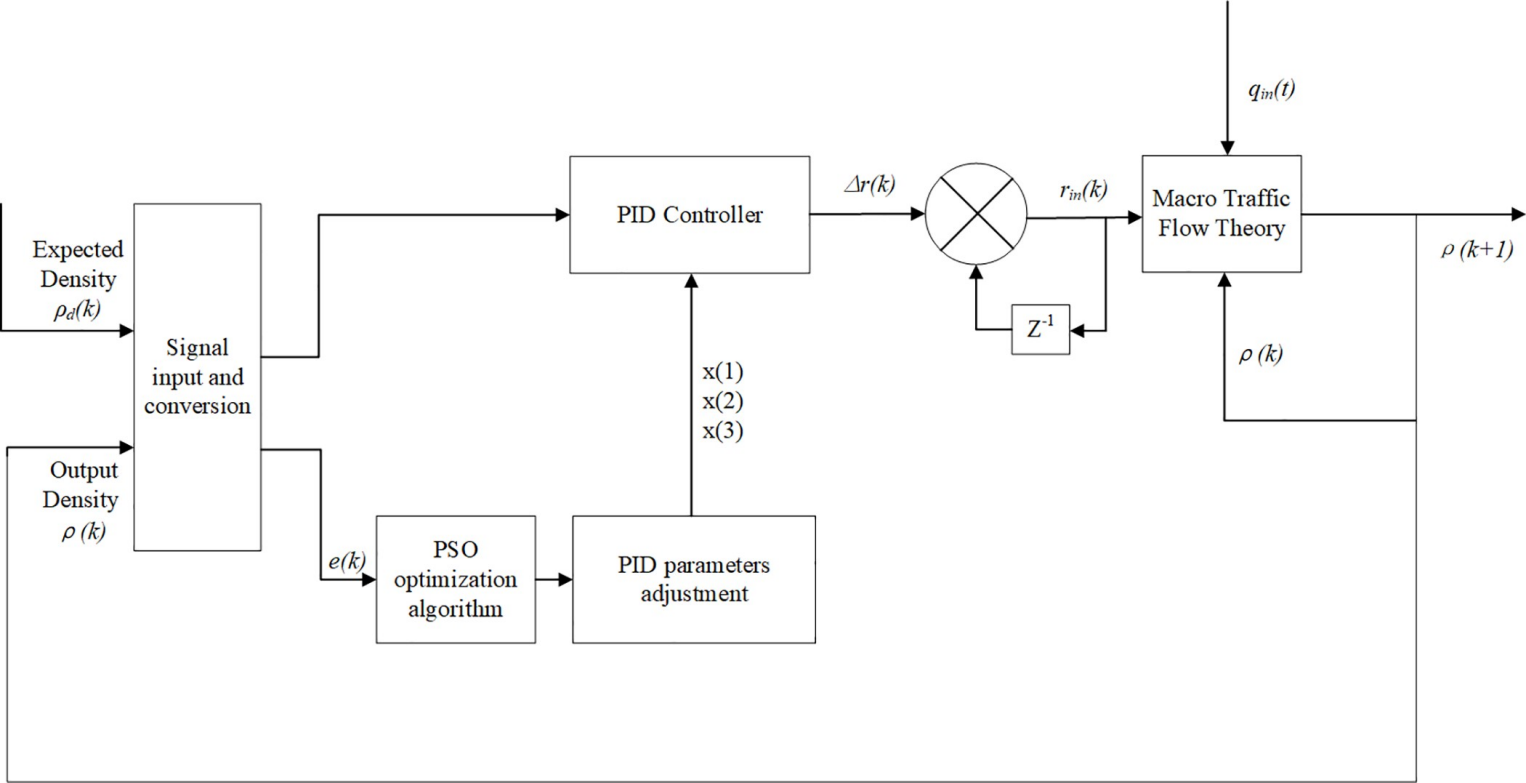
Flow chart of PSO-PID control system.

Different with the positional PID control, the incremental PID control does not need to accumulate *e(k)*, the state is only related to the previous state, and has little action error, so a better control effect can be achieved in control. When PSO generates a particle swarm, the particles in the group are assigned to the parameters *K*_*p*_, *K*_*i*_, and *K*_*d*_ in the PID controller at once, so the density performance index is obtained by running the system. Transfers the difference between output density and expected density to PSO as the adaptive value of the particles, and finally judges whether the algorithm can be exited. The specific process of PSO-PID control system is shown in Fig 2 flow chart of PSO-PID control system.

As can be seen from Fig 2 above, the PID control system is a closed loop feedback regulation system. In the initial state, the density is assigned to expected density*ρ*_*d*_*(k)*, and the *P*, *I*, and *D* parameters are determined by the PSO optimization algorithm. *e(k)* is the error signal, when the error occurs, the controller will take effect immediately to reduce the error and meet the requirements. The PID control output is increment *Δr(k)* which is converted into control variable ramp regulation rate *r(k)*, and put *r(k)* into the macro traffic flow model. The input flow *q*_*in*_*(t)* is known as external disturbance variable, and the output variable is state density*ρ(k+1)* according to the road detector. *Z* is the transfer function. This density then be fed back to the controller and compared with the expected density in order to re-adjust the PID controller parameters. The process cycles back and off until it converges. After optimization in the program, the input of PID controller is as follows:

x(1)=error(k)−error_1 (8)

x(2)=error(k) (9)

x(3)=error(k)−2error_1+error_2 (10)

Here, *error _ 1* and *error _ 2* respectively represent the difference error in the state of *k-1* and *k-2*. The output of the incremental PID controller is:

$$\Delta r(k)=\Delta u(k)=K_{p}x(1)+K_{I}x(2)+K_{D}x(3)$$ (11)

In the control system shown in Fig 2, the actual traffic flow density can track the desired traffic flow density by adjusting the on-ramp regulation rate. This feedback control can suppress the model error and disturbance input noise of expressway.

Assuming a population composed of N particles in a D-dimensional search space, in which the *i* particle represents a D-dimensional vector *X*_*i*_ = (*X*_*i*1_, *X*_*i*2_,⋯,*X*_*iD*_)^*T*^ and represents a potential solution of the problem. The fitness value corresponding to the position *X*_*i*_ of each particle can be determined by the objective function. The velocity of particle *i* is (*V*_*i*1_, *V*_*i*2_,⋯,*V*_*iD*_)^*T*^, the individual extreme value is *P*_*i*_ = (*P*_*i*1_, *P*_*i*2_,⋯,*P*_*iD*_)^*T*^, and the population extreme value is *P*_*g*_ = (*P*_*g*1_, *P*_*g*2_,⋯,*P*_*gD*_)^*T*^. In the iterative cycle process, the particle updates the velocity and position information through its extreme values *P*_*i*_ and *P*_*g*_, that is:

$$V_{id}^{k+1}=\omega V_{id}^{k}+C_{1}R_{1}(P_{\mathrm{id}}^{k}-X_{id}^{k})+C_{2}R_{2}(P_{\mathrm{gd}}^{k}-X_{id}^{k})$$ (12)

$$X_{id}^{k+1}=X_{id}^{k}+V_{k+1,id}$$ (13)

Here, *ω* is the inertia weight; *d* = 1,2,3…*D*; *i* = 1,2,3…*n*; k is the current number of iterations; $V_{id}^{k+1}$ is the velocity of particles under the current number of iterations; *C*_1_, *C*_2_ is the acceleration factor, and *C*_1_, *C*_2_∈*R*^+^; *R*_1_, *R*_2_ is a random number distributed in the interval [0,1].

## 4. Case study

### 4.1 Single ramp metering control simulation

#### 4.1.1 Case scene and parameters

**Fig 3:**
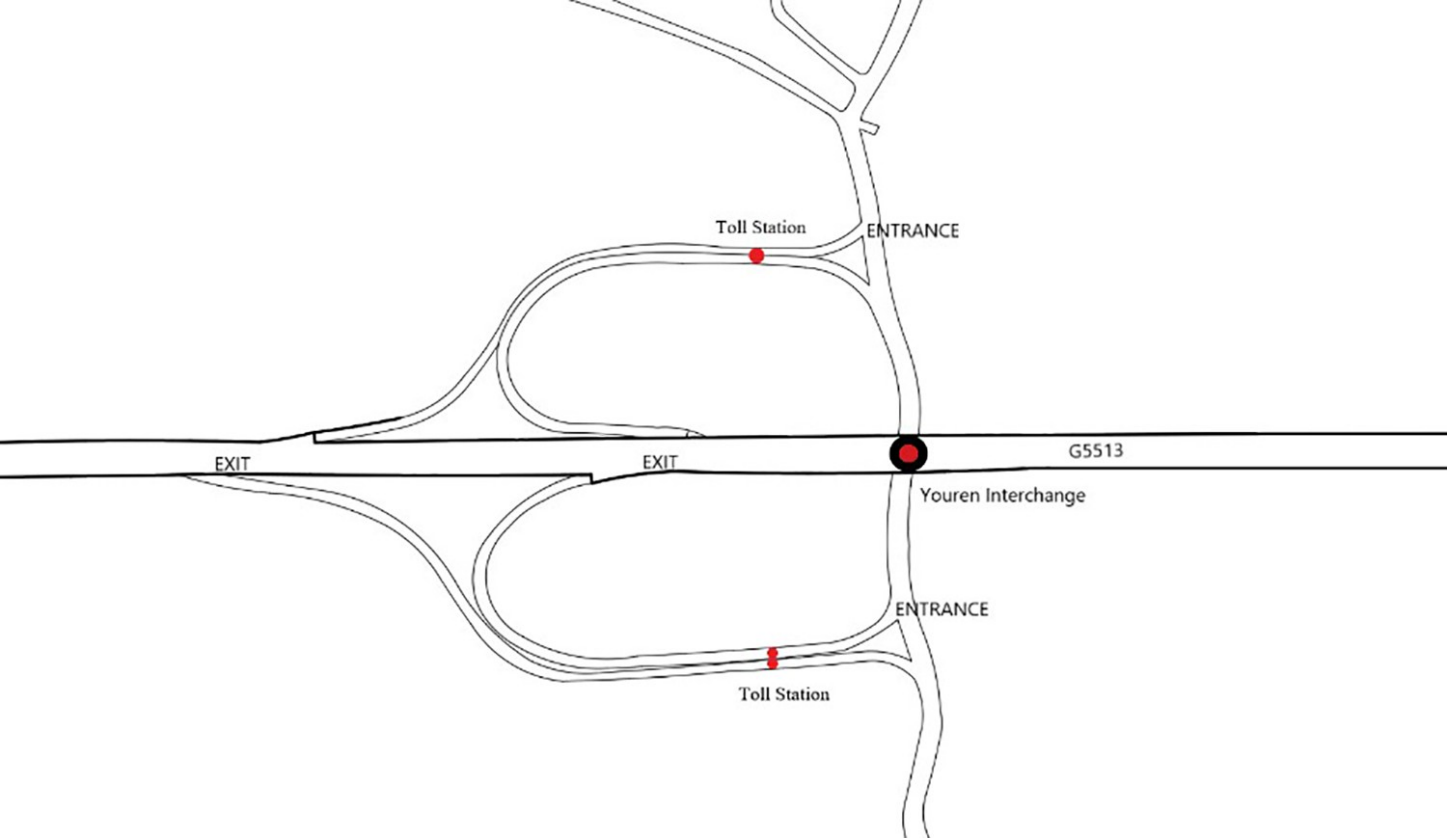
Layout of Youren Interchange.

Hunan Changyi Freeway (G5513) is a two-way four lane freeway with a design speed of 100km/h. This freeway starts from Changsha West toll station and ends at Yiyang North toll station. Seven toll stations are set along the route, with a total length of 62 km and an average travel time of 38 minutes. It intersects with hubs of Changxiang Freeway and Yiyang South Freeway. A total of 8 main line traffic detection stations are set up across the line. Youren Interchange in Changyi Freeway between Changsha West and Yiyang North is taken as a case study to establish a road network model, Fig 3 is the layout of Youren Interchange. It is always congested during holidays and daily peak hours.

According to main line data of traffic station investigation and every 5 minutes running data from toll station in April, the 13 hours traffic data from 5: 00 A.M. to 17: 00 P.M. during the Qingming Festival on April 5 are analyzed, as shown in Table 1 below:

**Table 1:** Traffic flow at Changyi Freeway.

Time	Traffic (pcu/h)	Time	Traffic (pcu/h)
**5:00**	2639	12:00	3081
**6:00**	3142	13:00	3070
**7:00**	2780	14:00	3148
**8:00**	2739	15:00	3095
**9:00**	2623	16:00	2532
**10:00**	2390	17:00	2650
**11:00**	3037		

**Fig 4:**
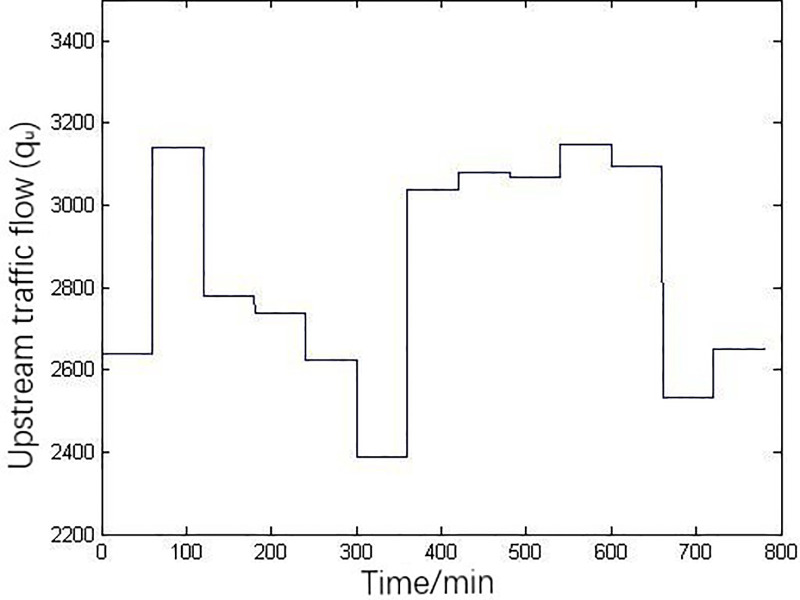
The actual flow variation input of upstream.

**Fig 5:**
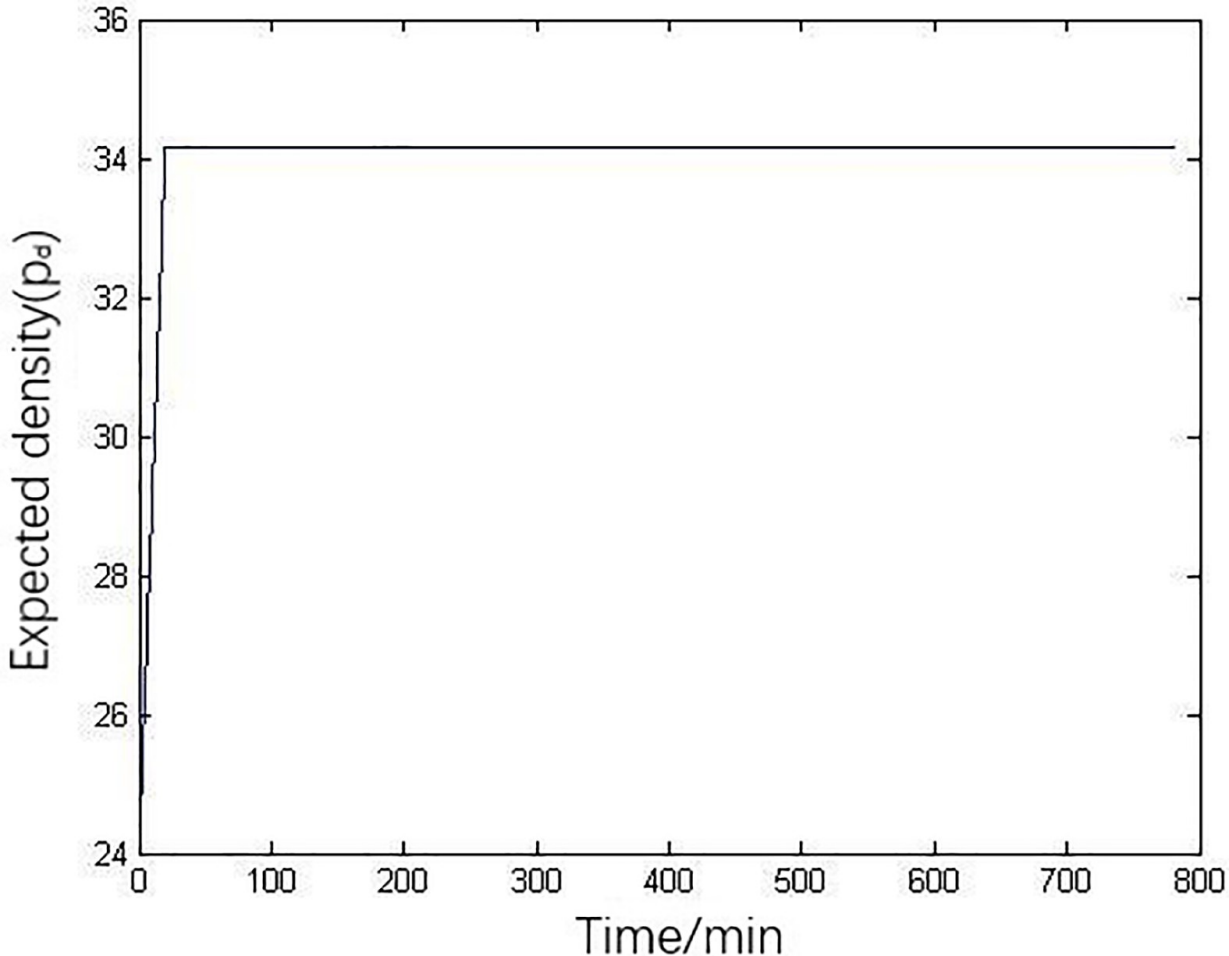
The expected density diagram.

The simulation parameters in the model are set as follows: number of lanes is *λ* = 2, free-flow speed is *V*_*f*_ = 97.3km/h, jam density is *ρ*_*jam*_ = 74 vehicles/km/lane, critical density is *ρ*_cr_ = 36.14 vehicles/km/lane, and simulation time step is set as *Δt* = 20 seconds. Fig 4 is the actual flow variation input of upstream in Youren Interchange, which swells and subsides. In order to prevent the mainline traffic from congestion, we should keep the density of mainline traffic flow as the considerable value, listed as the expected density, Fig 5 is the expected density diagram.

#### 4.1.2 Results and discussion

**Fig 6:**
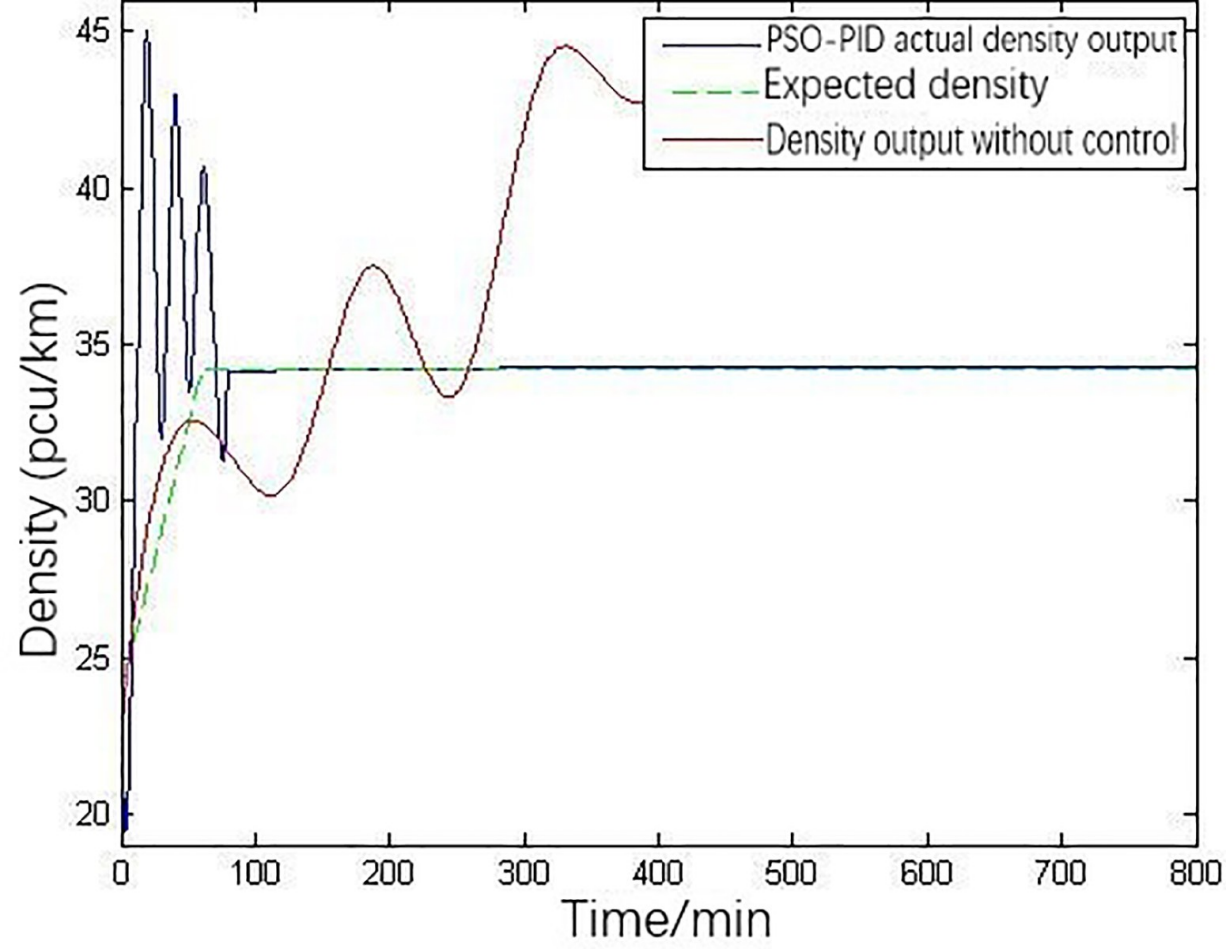
Density variation under PSO-PID control.

**Fig 7:**
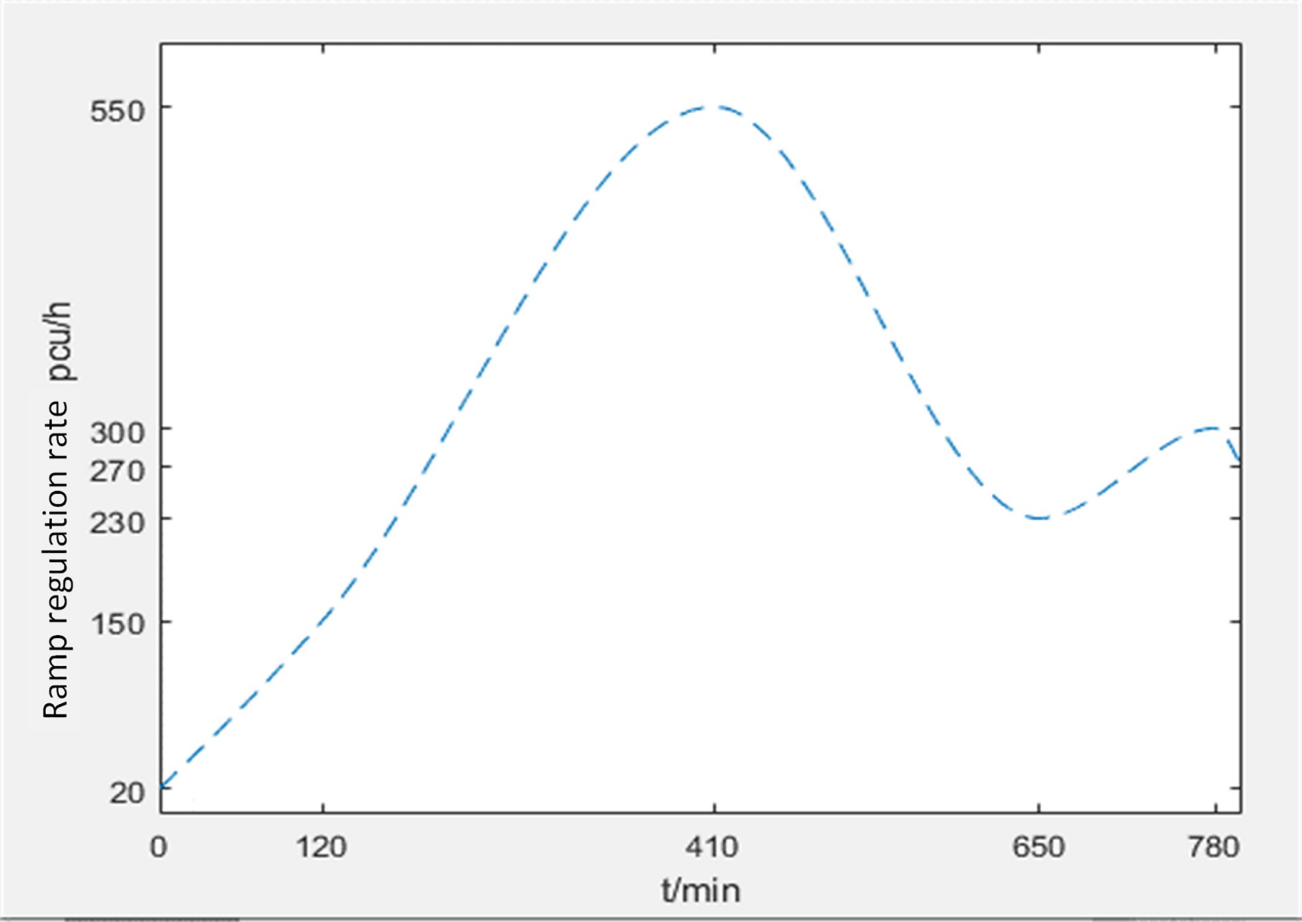
The curve of ramp metering.

The simulation result of density variation under PSO-PID control based on MATLAB platform is shown in Fig 6 with the same data and parameters in Fig 4, which gives the density variation of downstream section under PSO-PID control. The green dotted line represents the expected density, the red line represents the density without control, and the blue line represents the density under PSO-PID control. Fig 7 is the curve of ramp metering under PSO-PID control.

In the absence of control measures, after about 300 minutes, the main line density rises steadily and forms a long period congestion. If there is no intervention, the congestion will inevitably increase and spread to the upstream. The overall control system tends to converge at approximately 80 minutes after 240 iterations. Due to the large traffic flow input, the parameters need to be preliminarily iterated, which will cause density shake obviously and make the ramp control system in an unstable state in the initial stage of ramp metering. Due to the large traffic flow input, the parameters need to be preliminarily iterated, which will cause density shake obviously and make the ramp control system in an unstable state in the initial stage of ramp metering. However, with the real-time control and traffic flow adjustment, the input is more and more stable, the PID control parameters are gradually stable, as well as the freeway traffic flow. The ramp entry rate can be controlled in real time based on the real-time upstream traffic flow data. Meanwhile, by learning from PSO algorithm, the PID parameters are changing in real time, which reflects the significance of intelligent traffic management in freeway ramp metering. The result shows that the PSO-PID ramp metering has a good effect on the density control.

**Fig 8:**
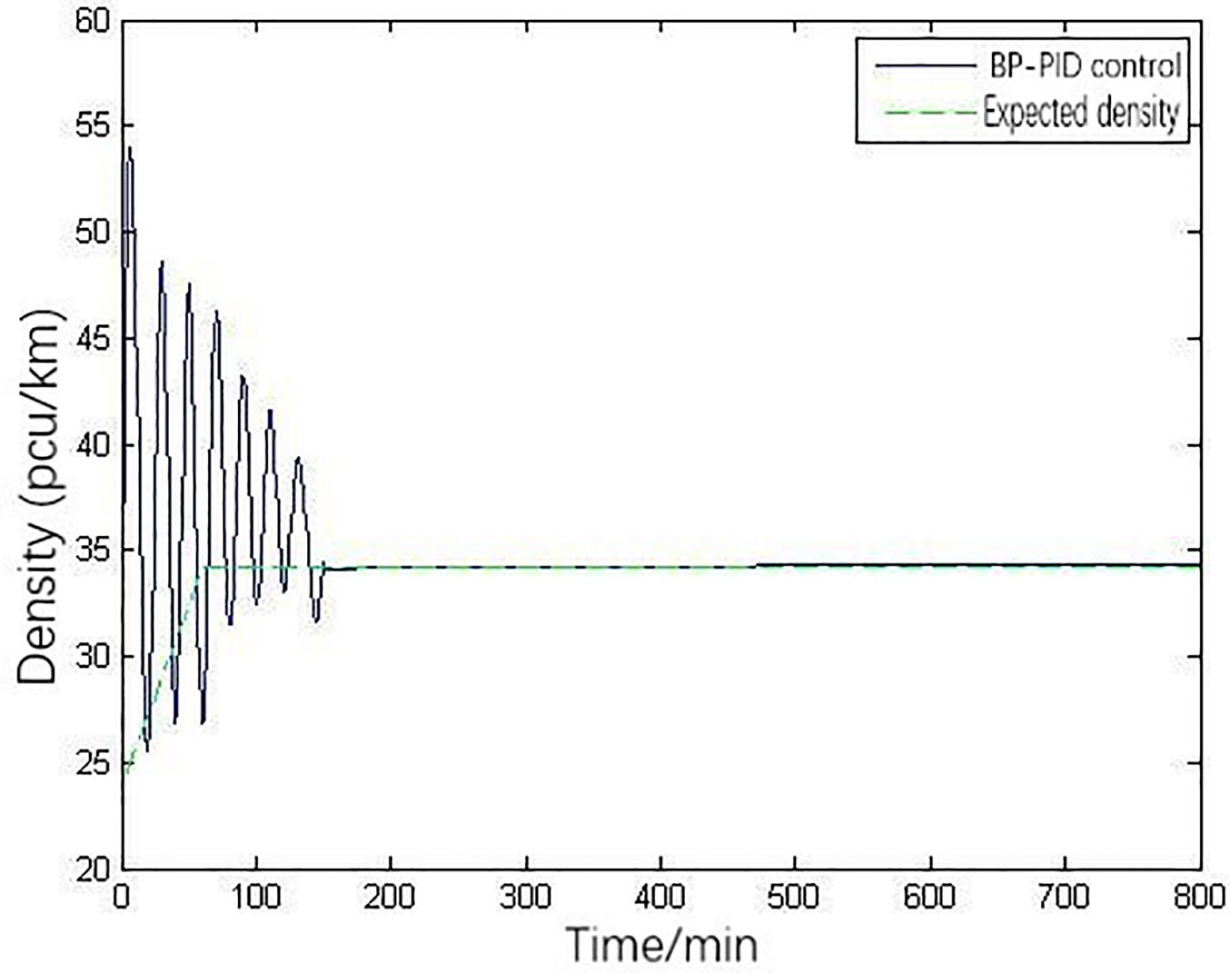
Density variation under BP-PID control.

The PSO is compared with the BP neural network in order to show the advantages of PSO. The dimension of the input and output layer in BP neural network is completely determined according to the operators’ requirements. The three parameters are the output of on-ramp metering, so the output layer will have three nodes. The control quantity, i.e., the regulation rate of the on-ramp, is obtained through progressive propulsion. It should be noted that the input quantity must be detected and has a great impact on the output quantity. In addition, it is also required that each input variable has an independent relationship with each other. The parameters adopted here are the same input flow data and system parameters. Fig 8 is the control result of the main line section in ramp control density based on BP neural network below.

It can be seen that the PID ramp metering system using BP neural network tend to converge in about 150 minutes after 450 iterations. Compared with PSO, the convergence speed of BP neural network is 70 minutes slower, and the number of iterations is 210 times more. The density with PSO is closer to the given ideal density, and the oscillation degree is obviously less than that of BP neural network.

## 5. Conclusion

According to the running data collected by toll station and profile detectors, the intelligent PID control is proposed based on macro traffic flow model, and it is optimized by the PSO for the single ramp metering. It can achieve the control goal of smooth traffic flow on main line section near on-ramp and keep the density near the expected value all the time. An example simulation experiment based on the MATLAB is carried out in the Youren Interchange of Changyi Freeway. The results show that the PSO-PID control system has rapid convergence speed and better effect. With the PSO-PID control of ramp traffic flow, the main traffic flow becomes stable, and the freeway tends to be stable simultaneously. As a comparison, the paper uses BP neural network to optimize the PID control parameters in the same simulation, the result shows that the convergence speed of PSO-PID control is faster than the BP neural network-PID, but there is little difference in convergence accuracy. The service level and actual density of the freeway main line and on ramp can keep in an ideal range by adopting the PSO-PID control. Moreover, the traffic flow rate and traffic safety of expressway can be improved, and the total travel time and the accident rate during peak period can be reduced. Furthermore, the ramp control not only can optimize the travel efficiency of the main sections, but also for the connecting urban roads at the exit ramp.

The most obvious practical difficulty in implementing our approach is the data accuracy issue for freeway operators since it is often the case that the detectors on the mainline are spares and the data collection usually takes a long-time interval. To solving this problem, the transportation authority could take the lead and make the real time data collection possible. For future research, we are interested in extending the developed model to integrate both single and multi-ramp metering in order to obtain an optimal traffic flow input allocation during the holidays and peak hours.

## Supporting information

S1 Dataset (XLSX)

## References

[1] Zheng J., Dong D., Chen H, Comparative analysis of on-ramp control strategies for urban expressways. Computer Measurement and Control, 2006, 14 (2): 196.

[2] Wattleworth J A, Berry D S, Peak period analysis and control of a freeway system—some theoretical investigations. HRB Rec, 1965, 89: 1.

[3] Ren L, Summary of freeway on-ramp control methods. Traffic Standardization, 2006 (5): 146.

[4] Papageorgiou M A new approach to time-of-day control based on a dynamic freeway traffic model. Transportation Research, Part B, 1980, 14:349–360.

[5] Papageorgiou M, Hadj-Salem H, Blosseville J M, ALINEA: A local feedback control law for on-ramp metering. International Conference on Road Traffic Control. IET, 1991, 194–198.

[6] Papageorgiou M, Freeway Ramp Metering: An Overview. Dearborn (MI), USA: 2000.

[7] Smaragdis E, Papageorgiou M, Kosmatopoulos E. A, Flow-maximizing adaptive local ramp metering strategy. Transportation Research Part B: Methodological. 2004, 38(3), 251–270.

[8] Taylor C, Meldrum D, Jacobson L, Fuzzy ramp metering-design overview and simulation results. Transportation Research Record, 1998, 16(34):10–18.

[9] Zhang H M, Freeway ramp metering using artificial neural network. Transportation Research, Part C, 1997, 6(5)::273–286.

[10] Stephanedes Y J, (1994). Implementation of online zone control strategy for optimal ramp metering in the Minneapolis ring road. International Conference on Road Traffic Monitoring & Control. IET, 181–184.

[11] Liu J C, Kim J, Lee S, The advanced distributed ramp metering system (ARMS). Workshop on Parallel & Distributed Real-time Systems, 1994.

[12] Kotsialos A, Papageorgiou M, The Importance of Traffic Flow Modeling for Motorway Traffic Control. Networks and Spatial Economics, 2001; 1(1–2):179–203.

[13] Kotsialos A, Papageorgiou M, Mangeas M, Coordinated and integrated control of motorway networks via non-linear optimal control. Transportation Research Part C (Emerging Technologies), 2002; 10(1):65–84.

[14] Paesani G.F, System wide adaptive ramp metering in southern California. ITS America 7th Annual Meeting and Exposition: Merging the Transportation and Communications Revolutions. 1997.

[15] Laing X.R, Liu Z.Y, Mao Z.R, Design of nonlinear feedback ramp controller in freeway and its simulation. Computer Engineering and Applications, 2005; 41(020):111–113.

[16] Bellemans T, Schutter B D, Moor, B D, Model predictive control for ramp metering of motorway traffic: A case study. Control Engineering Practice, 2006; 14(7)::757–767.

[17] Zeng A.G, Laing X.R, Wei Y.X,. Freeway ramp PID controller regulated by RBF neural network, Computer Engineering and Applications, 2007; 43 (36): 189–191.

[18] Bellemans T, Schutter B, Wets G, Model predictive control for ramp metering combined with extended Kalman Filter-Based Traffic State Estimation. Intelligent Transportation Systems Conference. IEEE, 2006; 406–411.

[19] Papamichail I, Papageorgiou M, Vong V, Heuristic ramp-metering coordination strategy implemented at Monash Freeway, Australia. Journal of the Transportation Research Board, 2010; 2178: 10–20.

[20] Gokasar I, Ozbay K, Kachroo P, Coordinated feedback-based freeway ramp metering control strategies “C-MIXCROS and D-MIXCROS” that take ramp queues into account. Advances in Dynamic Network Modeling in Complex Transportation Systems. Springer New York. 2013.

[21] Kotsialos A, Margonis I, Coordinated ramp metering for freeway networks–A model-predictive hierarchical control approach. Transportation Research Part C Emerging Technologies, 2010; 18(3):311–331.

[22] Fares A, Gomaa W, Freeway ramp-metering control based on Reinforcement learning. IEEE International Conference on Control & Automation. IEEE, 2014; 1226–1231.

[23] Fares A, Gomaa W, Multi-agent reinforcement learning control for ramp metering. Progress in Systems Engineering. Springer International Publishing, 2015.

[24] Meshkat A, Zhi M, Vrancken J L M, Coordinated ramp metering with priorities. Intelligent Transport Systems IET, 2015; 9(6)::639–645.

[25] Jeon S., Jung I, Coordinated ramp metering for minimum waiting time and limited ramp storage. IEICE Transactions on Fundamentals of Electronics, Communications and Computer Sciences, 2016; 99(10):1843–1855.

[26] Belletti F, Haziza D, Gomes G, Expert level control of ramp Metering based on multi-task deep reinforcement learning. IEEE Transactions on Intelligent Transportation Systems, 2017; 19(4):1198–1207.

[27] Ivanjko E, Koltovska D, Greguri M, Ramp metering control based on the Q-Learning algorithm. Cybernetics and Information Technologies, 2015; 15:88–97.

[28] Gao K, Han F, Dong P, Xiong N, Du R, Connected Vehicle as a Mobile Sensor for Real Time Queue Length at Signalized Intersections. Sensors 2019, 19(9), 2059. doi: 10.3390/s19092059 31052585 PMC6538986

[29] Long K, Lin Q, Gu J, Wu W, Han L D, Exploring Traffic Congestion on Urban Expressways Considering Drivers’ Unreasonable Behavior at Merge/Diverge Sections in China. Sustainability 2018, 10, 4359.

[30] Wu W, Liu W, Zhang F, Dixit V, A New Flexible Parking Reservation Scheme for the Morning Commute under Limited Parking Supplies, Networks and Spatial Economics, 2021, 1–33.

[31] Wu W, Zhang F, Liu W, Lodewijks G, Modelling the traffic in a mixed network with autonomous-driving expressways and non-autonomous local streets, Transportation Research Part E: Logistics and Transportation Review 2020, 134(1):1–22.

[32] Zhao J, Ma W, Liu Y, Han K. Optimal operation of freeway weaving segment with combination of lane assignment and on-ramp signal control. Transportmetrica A-Transport Science, 2016, 12(5): 413–435.

[33] Zhao J, Knoop V L, Wang Me. Two-dimensional vehicular movement modelling at intersections based on optimal control. Transportation Research Part B: Methodological, 2020, 138, 1–22.

[34] Ji Zhao, Ma W, Xu H. Increasing the capacity of the intersection downstream of the freeway off-ramp using presignals. Computer-Aided Civil and Infrastructure Engineering, 2017, 32(8): 674–690.

[35] Long K, Yao W, Gu J, Wu W, Han L D, Predicting Freeway Travel Time Using Multiple- Source Heterogeneous Data Integration. Appl. Sci. 2019, 9, 104.

[36] Chen F, Chen S, Ma X, Analysis of hourly crash likelihood using unbalanced panel data mixed logit model and real-time driving environmental big data. Journal of Safety Research, 2018; 65:153–159. doi: 10.1016/j.jsr.2018.02.010 29776524

[37] Chen F, Chen S, Injury severities of truck drivers in single- and multi-vehicle accidents on rural highway, Accident Analysis and Prevention, 2011; 43:1677–1688. doi: 10.1016/j.aap.2011.03.026 21658494

[38] Dong B, Ma X, Chen Feng, Chen Suren, Investigating the Differences of Single- and Multi-vehicle Accident Probability Using Mixed Logit Model, Journal of Advanced Transportation, 2018(PT 1): 11.1–11.9.

